# Plasma Assisted Decontamination of Bacterial Spores

**DOI:** 10.2174/1874120700802010036

**Published:** 2008-07-25

**Authors:** Spencer P Kuo

**Affiliations:** Department of Electrical and Computer Engineering, Polytechnic University, Brooklyn, NY, USA

## Abstract

The efficacy and mechanism of killing bacterial spores by a plasma torch is studied. Bacterial-spore (*Bacillus cereus*) suspension is inoculated onto glass/paper slide-coupons and desiccated into dry samples, and inoculated into well-microplate as wet sample. The exposure distance of all samples is 4 cm from the nozzle of the torch. In the experiment, paper slide-coupon is inserted inside an envelope. The kill times on spores in three types of samples are measured to be about 3, 9, and 24 seconds. The changes in the morphology and shape of still viable spores in treated wet samples are recorded by scanning electron and atomic force microscopes. The loss of appendages and exosporium in the structure and squashed/flattened cell shape are observed. The emission spectroscopy of the torch indicates that the plasma effluent carries abundant reactive atomic oxygen, which is responsible for the destruction of spores.

## INTRODUCTION

Bacteria of various *Bacillus* species produce a dormant or inactive cell type called spore in response to nutrient-poor conditions [1, 2]. The spore consists of the following main parts: appendages, exosporium, outer coat, inner coat, cortex, and core [3-9]. Each vegetative cell forms one spore and undergoes lysis after the sporulation process is complete. The spore grows exclusively in the mammalian host where spores germinate in the presence of rich conditions such as amino acids, sugars, adequate pH, water, and a favorable temperature [2, 10].

Dormant spores are able to survive for long periods in soils and thus account for the ecological cycle of the organism. They may survive as long as 10 years in milk, 40 years in soil, 75 years in silk thread, 200 years in bones. The longevity of spores in the environment is an important factor in the epidemiology of anthrax and explains the predominant occurrence of the disease in herbivores. *B. anthracis*, the causative agent of anthrax, gram-positive, facultatively anaerobic, rod-shaped endospore-forming bacterium of the genus *Bacillus* that causes disease in humans and herbivore animals [11, 12]. *Bacillus cereus, Bacillus anthracis, Bacillus thuringiensis*, along with *Bacillus mycoides*, belong to the phylogenetically similar* B. cereus* group [13, 14]. *B. anthracis* exhibits genetic similarities with *B. cereus* [13, 14]. Indeed many consider them to be the same species [15].

Due to the highly fatal nature of pulmonary anthrax (80-90%), the ease of production and storage of the spores of *Bacillus* and their survival in the environment after bioattack, this organism (Anthrax) has become the primary bacterial agent in biowarfare and bioterrorism [16-18], for instance, used in the terror attacks of 2001 that have brought the issues surrounding the deliberate release of BWA into sharp focus. To counter the threat of terrorist attacks, an effective decontamination defense is required to minimize the consequences of biological attacks.

The traditional decontamination methods for BWA involve the use of “wet” solutions, which include bleaches and Decontamination Solution #2 (DS2). The decontamination time of these methods is typically around 30 minutes. A disadvantage with wet methods is that the current decontamination chemicals are corrosive to materials such as metals, plastics, rubber, paint, leather, and skin. Thus they are not suitable for the use on sensitive equipment. Moreover, the hazardous chemicals need to meet special guidelines for storage, transport, and disposal during and after usage because these chemicals could be released into the environment.

Alternative dry methods are being developed for many reasons, which include easily transported, fast working, no mass storage requirement, safe to personnel and inert to sensitive equipment. However, spore is highly resistant to a variety of treatments including ultraviolet, pressure and heat [4, 19-21]. Its coats shield the core from UV radiation; far UV radiation might control the rate of genetic inactivation of isolated microorganisms, but it could not sterilize the aggregated colonies thoroughly and thus UV treatment has little effect on the spore and leaves the spore’s immunology almost unchanged [22]. The membranes enable the spore to endure high pressure (100 - 200 Mpa). Low water content in the core makes the spore heat resistant. The efficiency of thermal energy method is also limited by the temperature constraint on avoiding damage to equipment and surfaces. Consequently, this method is relatively time-consuming. Moreover, spores are most refractory to inactivation by the boiling water method [23-25], which takes about 12 minutes to destroy *B. anthracis* spores [24]. Stein and Rogers [25] reported that vigorous boiling could reduce the time to within 3 to 5 minutes to destroy spores from 43 strains of *B. anthracis*. Boiling water in a covered vessel killed spores of the *Bacillus*, reducing the spore population by more than four orders of magnitude in 3 to 5 minutes. Holding water at a rolling boil could further reduce the time to about 1 minute to inactivate waterborne pathogens, including encysted protozoa [26, 27]; however, it would not inactivate the spores even by increasing the boiling time to 3 minutes as an open container was used. Ionizing radiation γ-ray is effective to decontaminate a large area in a heavily shielded fixed facility, but it is not easily implemented in the field and can be destructive to sensitive equipment. Air plasma in a highly energized state contains radicals such as excited atoms and molecules. Reactive oxygen species (ROS) can destroy just about all kinds of organic contaminants by means of chemical reactions, which result to carbonyls and carbonyl adducts and eventually, convert these contaminants into carbon dioxide and water.

In the present work, a portable arc-seed microwave plasma torch (MPT) [28] was applied in the plasma decontamination experiments. This torch ran steadily under plain airflow.* B. cereus* (BC) was chosen as a simulant of the *B. anthracis*. The effects of plasma produced by this MPT on spores in dry and wet environment were studied. In addition to the ultimate effect of killing them [29-31], the changes on the morphological characteristics of the spores during the exposure to the plasma effluent were also observed by scanning electron microscope (SEM) and by atomic force microscope (AFM).

## EXPERIMENTAL PREPARATIONS

### Plasma Torch

A schematic of the torch device and the arrangement for the experiments are shown in Fig. (**1**). In the present experiments the airflow rate is 0.393 l/s and the sample is placed at 4 cm below the nozzle of the torch. The torch has a good size (a height of about 25 mm and a volume of about 6 cc).

The kill rates on three types of samples were measured and compared. The exposure times of 2 to 16 seconds were chosen for the dry samples on glass coupons and for the wet samples in 96-well microplates. It was examined before the experiments that the torch would not cause noticeable desiccation of water sample in a well, a same arrangement as the experiments. In the experiments with samples prepared on paper-coupons and placed inside envelopes, the exposure times were limited to be less than 10 seconds, assuring that the temperature inside the envelope was low (< 70 °C). Exposure times for the samples to the plasma torch effluent were recorded when the arc-seeded MPT was in full-operation with the magnetron running; the delay of the magnetron operation, typically between 1 to 2 seconds, was ascribed to the necessary heating time of its filament.

### Spores

B. cereus ATCC 11778 spores were chosen as a stimulant substitute for B. anthracis. These spores are facultative aerobes (oxygen is needed for growth) and like other members of the genus Bacillus can produce protective endospores; widely distributed in nature and cause various food spoilage. But these agents are relatively benign microorganisms having the minimum safety requirement (i.e., Biosafety Level 1 (BSL1)) and do not ordinarily cause fatal human disease. However, great care is still required in the preparation of and handling of B. cereus spores, during and after experiments. These bacterial spores can cause two distinct types of illnesses: 1) diarrhea with an incubation time of approximately 4 to 16 hours, and 2) an emetic (vomiting) illness with an incubation time of 1 to 5 hours. Spore handling was carried out under a Biological Safety Cabinet.

### Sample Preparations Prior to and After Exposure

#### 1 Prior to Exposure

##### (a). Dry Samples on Glass Slide-Coupons

Glass coupons were prepared by fixing a glass slide to the Organ Tissue Culture dish and exposing it to UV irradiation during 5 hours to prevent cross contamination. Dry samples were prepared by inoculating 30µl of a spore suspension onto the UV irradiated glass slide-coupons, thus forming a spot ~0.5cm in diameter on each glass slide. Later on, samples were desiccated during 12 hours.

##### (b). Wet Samples

Drops of bacterial-spore suspension, also 30µl each, were taken directly as wet samples, which were inoculated into the wells of 96-well microplates (the volume of each well is 300 µl).

##### (c). Dry Samples on Paper-Coupons

A fiber mixed (DuPont™ Tyvek) envelope was cut into 1 × 1 cm squares to host spore samples. Each paper-coupon was fixed to the Organ Tissue Culture dish and exposed to UV irradiation for 5 hours to prevent cross contamination. Samples were prepared by inoculating 30µl of a spore suspension onto each of the UV irradiated paper-coupons, which were then desiccated for 12 hours. Subsequently, each coupon was inserted into an envelope. Envelopes were sealed before being exposed to a plasma torch and one of those was kept as a control. Spores were in a *‘confined environment’*.

In these sample preparations, every sample contained about 10^6 ^spores before the treatment.

#### 2 Procedures of Processing the Samples after Plasma Treatment

##### (a). For Dry Samples on the Glass Coupons

Treated spores and debris were removed from the glass slides by means of extensive sonication using tissue culture water. The mixtures were serially diluted from 10^-1 ^to 10^-7^.

##### (b). For wet Samples in the 96-Well Microplates

Volumes of solutions of untreated and post-exposed spore samples were checked to make sure that the 30µl dripped initially into each well of the plate was not reduced by more than 5% (controlled by the exposure distance and time). The post-exposure sample-handling was performed as follows: spores and debris in each well of the plate were diluted using W3500 tissue culture water (60ml/well) and mixed by means of extensive continuous shaking for 1 hr at 25 °C. The mixtures of post-exposed samples were serially diluted from 10^-1 ^to 10^-5^.

##### (c). For samples on the Paper-Coupons

Treated paper-coupons hosting spores and debris were placed into Organ Tissue Culture dishes. Then, 1 ml of tissue culture water was added to each dish. Soon after, each dish with coupon was extensively sonicated for 20 minutes at 20 °C with the purpose to remove unbound spores and debris from the paper-coupon. The mixtures were serially diluted from 10^-1 ^to 10^-7^.

In all cases, those diluted mixtures were plated onto petri dishes with Tryptic Soy (TS) liquid media and incubated at 37°C for 16 hours. Untreated control was sonicated and handled in the same manner as samples. To ensure full spore removal after sonication, only paper coupons were placed onto Petri dishes with TSA media, and then incubated. After the incubation, the resulting colony forming units (CFU) could be observed through their images in the taken pictures. Only the mixtures with 10^-1^, 10^-2^, and 10^-3^ dilution in the case (a), (b), and (c), respectively, were chosen for the analysis. The results of the counted CFU then compared with the control CFU (about 10^5^, 10^4^, and 10^3 ^per diluted sample before the treatment in the case (a), (b), and (c), respectively) to determine the decontamination efficacy (i.e., the survival curves or called “kill curves” in some literatures). No growth was observed on paper coupons.

## DECONTAMINATION EXPERIMENTS AND RESULTS 

### (a). Dry Samples on Glass Slide-Coupons

The capacity of the plasma torch on killing *B. cereus* spores is demonstrated in Fig. (**2**), which contains images representing the results of 10^-1^ diluted treated samples. As shown, almost all spores were killed in 12 seconds.

The CFU counts N of the experimental results (each set of experiments was run twice) were normalized to the initial number N_0_. These data points (Δ) are presented in Fig. (**3**) and are fitted by a straight line as the kill curve for dried B. cereus spores exposed to the plasma torch effluent at an exposure distance of 4 cm.

In Fig. (**3**), the x-axis represents exposure times in seconds and the y-axis displays the log of the ratio of the number of viable spores remaining (N) to the CFU control number (N_0_). The percentage of total spore remaining is plotted in the range down to -5 logs. The kill time, i.e., the time reduces the viable (BC) spore population by a factor of 10, is calculated to be about 3 seconds. For comparison, the ‘hot gas (175^0^ C)’ kill time [32] on the *Bacillus globigii* (BG) spore is about 45 seconds.

### (b). Wet Samples

The line fitting the data points (○) presented in Fig. (**3**) [33, 34] represents the kill curve for *B. cereus* spores in water exposed to the plasma torch effluent. The percentage of total spore remaining is plotted in the range down to -1 log. The kill time is calculated to be about 24 seconds, which is longer than the longest exposured time of 16 s in the experiment. Experiments with increasing exposure time to achieve many logs (> 3) of killing will be needed to obtain a more precise kill time.

### (c). Sample Contained Inside an Envelope

The capability of the plasma torch on killing of bacterial spores contained inside an envelope was tested. It was found [35] that the plasma effluent of the torch killed about 85% of spores in 7 seconds.

The CFU counts N from the experimental results were normalized to the initial number ${\text{N}}_{0}≅1000.$ These data points (х) are also presented in Fig. (**3**) and are fitted by a straight line as a kill curve. The percentage of total spore remaining is plotted in the range down to -1 log. The kill time is calculated to be about 9 seconds. A more precise value can be determined by experiments with increasing exposure time to achieve many logs (> 3) of killing.

## MORPHOLOGICAL STUDIES

In the wet-sample experiments, many viable spores still remained after the treatment. Thus the treated wet samples (of 10^-2^ dilution) with 3 cm exposure distance were used in the SEM and AFM studies [36] discussed in the following. SEM produces a two-dimensional (2-D) image to reveal the actual shape and the morphological structure of a bacterial spore, while AFM examines spore’s cell properties by taking a three-dimensional (3-D) image.

### 1 Scanning Electron Microscopy (SEM)

Solutions of untreated (10^4^ CFU) and post-exposed spore samples for SEM observations were deposited on mica disks and desiccated for 7 days.

Samples were then coated with a 10 nm thin film of evaporated gold [37] for 60 s and then observed with a SEM at an accelerating voltage of 20 kV. The images of untreated (Fig. **4a** in column A) and exposed but still viable *B. cereus spores* (Fig. **4a** in column B) were taken at high magnification for examination. Comparing two images reveals the changes of the actual shapes and morphological structures of the bacterial spore caused by the exposure. As shown, the spore has lost its appendages and exosporium and its size also decreases. However, the actual change of spore’s size has to be further checked by the corresponding 3-D images presented in the following section.

### 2 Atomic Force Microscopy (AFM) 

Solutions of untreated sample (10^4^ CFU) and those after exposure were immobilized on mica discs using sterile syringes, and then dried in air at 20^o^ C. All spores were found to be firmly attached to the mica disk and remained sufficiently bound to be imaged. Prepared samples were later mounted on an AFM sample holder for imaging. All AFM observations were carried out at 20^o^ C, using a Nano Scope^®^ IIIa controller as well as a MultiMode^TM^ microscope operating in tapping mode (amplitude) together with an E-scanner. A 125-µm silicon Nanoprobe was also employed. The calculated spring constant was 0.3 N/m. The resonance frequency remained in the range of 240-280 kHz, and the scan rate was 1 µm/s. Flattening and high-pass filtering of the image data were performed to remove the substrate slope from images and high-frequency noise strikes, which are, otherwise, more pronounced in the high-resolution tapping mode imaging.

3-D images of untreated *B. cereus* spores and a treated one from a wet sample exposed to the torch for 8 s, are presented in Fig. (**4b**) in columns A and B, respectively. It is noted that these images have different spatial scales. The axes on the horizontal plane extend to 3 ≥m. The vertical axis extends to 2 ≥m in Fig. (**4b**) of column A, and to 1 µm in the other. The images of untreated spores indicate that the basal membrane of *B. cereus* spore has an elongated shape and its cell has a bubbling shape in the middle region. The image also exhibits identifiable appendages (labeled by A) and is covered with a loose layer of exosporium (labeled by E) that has slightly spread and is stuck to the mica surface. After the exposure, the cell is squashed in the middle region and becomes elongated and wider. The middle part of the cell is flattened and the flattened region expands toward the two ends as seen in Fig. (**4b**) of column B.

AFM allows section analysis in virtual 3D. This provides accurate length (in third row labeled by (c)), width (in fourth row labeled by (d)), and height (in fifth row labeled by (e)) of an observed specimen. Section analysis (SA) reveals the approximate dimensions of the spores shown in the second row:

- Untreated spore: length 1.6 μm × width 1.1 μm × height 0.734 μm
- After 8 seconds treatment: 2.8 μm × width 1.8 μm × height 0.275 μm

Spore dimensions have changed significantly: a 175% increase in length (from 1.6μm to 2.8 μm), a 164% increase in width (from 1.1 μm to 1.8 μm), but a 267% decrease in height (from 0.734 μm to 0.275 μm). This morphology change can be best described as a structural depletion or collapse.

## PLAUSIBLE MECHANISM

A thermocouple (Omega model: DP460) was used to check the water temperature increase by the torch. This was performed with the water drop in a petri dish, rather than in a 96-well microplate (the probe of the thermocouple is too large for the well). Therefore, the volume of the water drop in the petri dish was larger than that in the well of the microplate. The temperature increase was negligibly small. Vaporizing less than 5% of the volume of the sample solution after the exposure further suggests that the gas temperature of the torch at the sample location could not be high. We have also exposed the thermo probe directly to the torch (at a distance ≥ 3 cm) without covering by a water drop; the temperature increase never exceeded 45° C. Moreover, we have placed a piece of paper slightly above the sample location (~ <4 cm) and found that the plasma torch could not even make a noticeable burn mark on the paper; however, it was also understood that the ignition point of paper is 233^0^ C. Therefore, it was necessary to measure the temperature inside the envelope in the exposed region. We placed the probe of the thermocouple inside an envelope at the distance ~ 4 cm below the torch exit as that of the experiments. The response time of the thermocouple is about 0.5 seconds. With 8 seconds of exposure to the torch, we found that the temperature was only raised to 38° C from the room temperature of about 26° C. We then increased the exposure time to 12 seconds; the temperature of the thermocouple was raised to 42.7°C. With 40 % duty cycle, the peak temperature increase would not be more than 17°C × 2.5 = 42.5°C. Therefore, the peak temperature should not exceed 70°C for 12 seconds exposure time and should be much lower than 70^°^ C in the case with 7 seconds of exposure to the torch. In any cases, the temperature was too low to introducing any possible killing effects on the spores. Therefore, thermal process as the decontamination mechanism is ruled out in all experiments.

Moreover, the recorded 2-D and 3-D images of the* B. cereus* bacterial spore show that before it is killed by exposing to the plasma effluent, its morphology is first destroyed and its cell is squashed and flattened. The observed change on spore´s shape and morphological structures evidences that it is the non-thermal mechanism responsible for the destruction of spores

The diameter of the circular nozzle exit of the torch on the cavity surface is about 1.25 cm, which is much smaller than the wavelength of about 12.3 cm. Hence, it is not likely the evanescent fields leaking out of the cavity could maintain significant amplitudes, even at the closest sample location (3 cm away from the exit hole), to directly interact with spores. For the safety reason, a microwave leakage detector (MD-2000) was used in experiments to monitor the level of the microwave flux. It was less than 1 mW/cm^2^ at 1 m distance away from the torch. 2.45 GHz microwave would be effectively absorbed by water in wet samples. However, the water temperature was not raised noticeably, this also evidenced that the microwave leakage could not be high. Thus we also rule out the possibility that leaked microwave radiation or the evanescent fields could be responsible for killing spores as well as for the changes on the morphological characteristics of the spore.

Oxidization of* B. cereus* bacterial spores by reactive radicals generated in the plasma effluent (such as O, NO and O_3_) or in water (such as OH•) is then considered to be the mechanism of killing spores in the experiments. Chemically reactive oxygen species (ROS) [4, 34-36, 39] are known to be effective in the destruction and annihilation of bacterial spores, and atomic oxygen is probably the most effective one among them in decontamination.

**Fig. (1):**
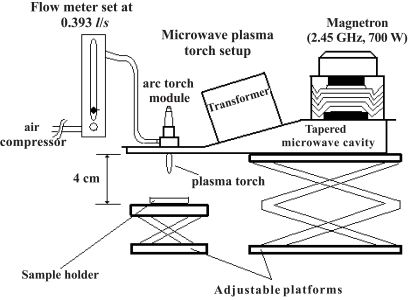
Schematic of a microwave torch device and the experimental arrangement.

**Fig. (4):**
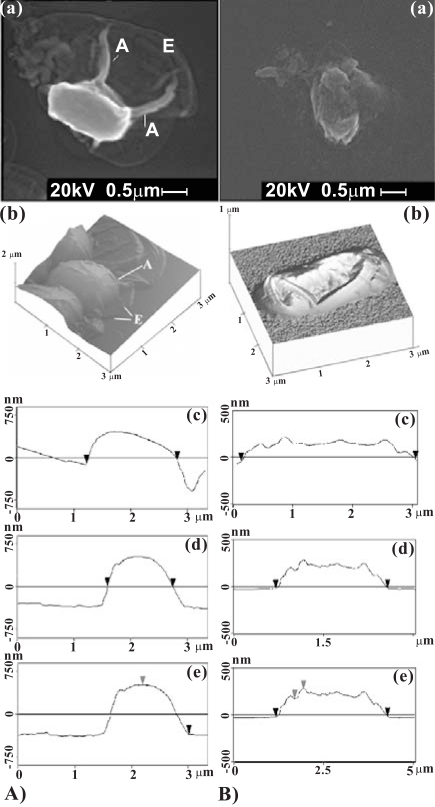
(**a**) SEM images (first row) and (**b**) AFM 3D images (second row), and cross section analyses (SA) for the (**c**) length, (**d**) width, and (**e**) height of an untreated *B. cereus* spore (column A) and of a treated and yet viable spore (column B) for 8 s.

Though the required energy to dissociate an oxygen molecule into two oxygen atoms is quite high; it needs about 5 eV electrons to effectively dissociate O_2 _into atomic oxygen [38]. However, air plasma produced by the present torch can effectively absorb microwave waves to reach a highly energized state, which can be the catalyst to produce atomic oxygen in the airflow. This is verified by the emission spectroscopy of the torch [28]. A triplet in the spectral region between 777.1 and 777.6 nm, as shown in Fig. (**5**), is the signature of atomic oxygen (O I) spectral lines. The emission spectrum in the UV range has also been checked; the spectral intensity including the OH• line at 305 nm is not strong, comparing with those of the O I lines. In this measurement, the emission is from a line of sight at 2 cm below the surface of the waveguide (shown in Fig. **1**).

The intense O I spectral lines shown in Fig. (**5**) indicate relatively high atomic oxygen content in the plasma effluent of the torch. An experiment was performed to examine if the atomic oxygen produced in the plasma effluent could penetrate into an envelope. A piece of paper from an envelope was hanging about 3 cm above the nozzle of the torch. Emission spectroscopy of the torch from a position slightly above the paper was measured. A comparison of the results obtained with and without a piece of paper blocking the torch flow indicated that about 40 % of the atomic oxygen passed through the paper.

However, the lifetime of the atomic oxygen is about 0.05 ms. With a flow speed of 20 m/s, most of the produced atomic oxygen can only survive a travel distance of about 0.1 cm. Therefore, the 4 cm exposure distance is reachable only by some of the atomic oxygen produced by the torch. On the other hand, ozone and nitric oxide (NO) [39] formed through the reactions of O and O_2_ and O and N, respectively, have a longer lifetime and can also be culprit.

It is difficult for atomic oxygen to penetrate into water. Although atomic oxygen can react with water to produce Hydrogen Peroxide (H_2_O_2_), H_2_O_2_ is not effective in killing spores. Therefore, a likely scenario is that the spores tend to float on the surface of the solution, such that the atomic oxygen produced by the MPT does not have to diffuse deeply into water to carry out decontamination. Again, ozone formed through the reaction of O and O_2_ can also become culprit. It is also noticed that the torch contains metallic particulates (Fe). Thus, another possibility is *via *OH• radicals formed in water through collision of metallic particulates with water molecules.

Thus the non-thermal destruction mechanism explaining the experimental results primarily involves the chemical reactions of ROS with nucleic acids, lipids, proteins and sugars. Active oxygen species and singlet oxygen are considered to cause extensive oxidative damage to biological macromolecules including lipids, carbohydrates, and proteins.

## CONCLUSION

The plasma effluent of the MPT carries abundant atomic oxygen. It is applied to kill spores *via* the oxidation mechanism. It is shown that the kill time (i.e., 10-fold reduction time) for the wet sample is about 24 seconds. This time is much longer than the corresponding one of 3 seconds for the dry sample [21], which is directly in contact with the plasma effluent. This is understandable because the ROS in the plasma effluent had to pass through a thin water barrier before reacting with the spores; and probably, the density of OH• radicals formed in water through collision of metallic particulates with water molecules was low. The kill time for the paper-coupon sample placed underneath the double-layered glue area of an envelope is about 9 seconds. It reduces to a half if only a single paper layer covers the sample. These results confirm that the MPT produces effective dry decontaminant, which is effective to kill virulent anthrax spores. The advantageous features of this decontaminant are summarized as

1. short kill time (~ 17 s for 6-log kill at a decontamination distance of 4 cm)
2. long decontamination distance (~ 4 cm)
3. large decontamination area (~ 1 cm2)
4. decontamination in wet environment
5. kill spores inside an envelope without damaging the envelope.

Applications of this technology include 1) air purifier and/or HVAC system, 2) medical sterilization facility for clinical pathogens, 3) mail decontaminator [40], and 4) decontamination of sensitive and delicate instruments, etc.

## Figures and Tables

**Fig. (2):**
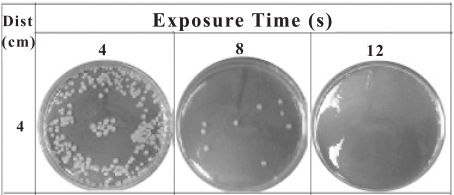
CFU formation after decontamination using plasma torch at a distance of 4 cm and exposure times of 4, 8, and 12 seconds.

**Fig. (3):**
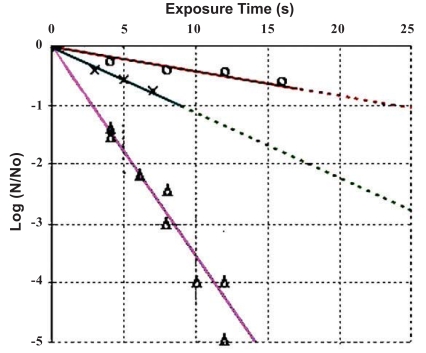
BC kill curves: data points (″Δ″ from dry samples on glass slide-coupons, ″x″ from samples on the paper-coupons, and ″o″ from wet samples) are obtained by placing samples at a exposure distance of 4 cm from the nozzle of a microwave plasma torch.

**Fig. (5):**
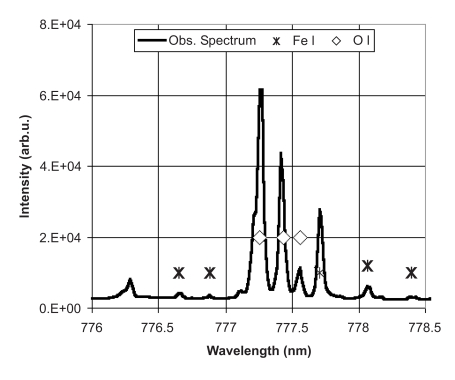
A part of a typical emission spectrum from the torch with atomic oxygen lines ^5^P(J=3)-^5^S°(J=2) at ~777.19 nm, ^5^P(J=2)-^5^S°(J=2) at ~777.42 nm, and ^5^P(J=1)-^5^S°(J=2) at ~777.54 nm.
